# Ganetespib selectively sensitizes cancer cells for proximal and distal spread-out Bragg peak proton irradiation

**DOI:** 10.1186/s13014-022-02036-z

**Published:** 2022-04-11

**Authors:** Simon Deycmar, Elisabeth Mara, Sylvia Kerschbaum-Gruber, Verena Waller, Dietmar Georg, Martin Pruschy

**Affiliations:** 1 Laboratory for Applied Radiobiology, Department of Radiation Oncology, University Hospital Zurich, University of Zurich, Winterthurerstr. 190, 8057 Zurich, Switzerland; 2 Department of Pathology, Section on Comparative Medicine, Wake Forest School of Medicine, Winston-Salem, USA; 3 Department of Radiation Oncology, Medical University of Vienna, Vienna, Austria; 4 University of Applied Science, Wiener Neustadt, Austria; 5 MedAustron Center for Ion Beam Therapy and Research, Wiener Neustadt, Austria

**Keywords:** Proton radiotherapy, Ganetespib, Linear energy transfer, Rad51, HSP90

## Abstract

**Objective:**

Hypersensitivity towards proton versus photon irradiation was demonstrated in homologous recombination repair (HRR)-deficient cell lines. Hence, combined treatment concepts targeting HRR provide a rational for potential pharmaceutical exploitation. The HSP90 inhibitor ganetespib (STA-9090) downregulates a multitude of HRR-associated proteins and sensitizes for certain chemotherapeutics. Thus, the radiosensitizing effect of HSP90-inhibiting ganetespib was investigated for reference photon irradiation and proton irradiation at a proximal and distal position in a spread-out Bragg peak (SOBP).

**Methods:**

A549 and FaDu cells were treated with low-dose (2 nM resp. 1 nM) ganetespib and irradiated with 200 kV photons. Proton irradiation was performed at a proximal and a distal position within a SOBP, with corresponding dose-averaged linear-energy transfer (LET_D_) values of 2.1 and 4.5 keV/µm, respectively. Cellular survival data was fitted to the linear-quadratic model to calculate relative biological effectiveness (RBE) and the dose-modifying factor (DMF). Additionally, A549 cells were treated with increasing doses of ganetespib and investigated by flow cytometry, immunoblotting, and immunofluorescence microscopy to investigate cell cycle distribution, Rad51 protein levels, and γH2AX foci, respectively.

**Results:**

Low-dosed ganetespib significantly sensitized both cancer cell lines exclusively for proton irradiation at both investigated LET_D_, resulting in increased RBE values of 10–40%. In comparison to photon irradiation, the fraction of cells in S/G2/M phase was elevated in response to proton irradiation with 10 nM ganetespib consistently reducing this population. No changes in cell cycle distribution were detected in unirradiated cells by ganetespib alone. Protein levels of Rad51 are downregulated in irradiated A549 cells by 10 nM and also 2 nM ganetespib within 24 h. Immunofluorescence staining demonstrated similar induction and removal of γH2AX foci, irrespective of irradiation type or ganetespib administration.

**Conclusion:**

Our findings illustrate a proton-specific sensitizing effect of low-dosed ganetespib in both employed cell lines and at both investigated SOBP positions. We provide additional experimental data on cellular response and a rational for future combinatorial approaches with proton radiotherapy.

**Supplementary Information:**

The online version contains supplementary material available at 10.1186/s13014-022-02036-z.

## Introduction

The superior depth dose distribution of proton beams relative to photon beams is the primary rationale for their clinical utilization [1]. However, the differential proton radiobiology and the biological factors involved remain widely unestablished and did not find clinical implementation so far. As a result, a generic relative biological effectiveness (RBE) factor of 1.1 is applied in present-day clinical treatment planning of proton radiotherapy. This paradigm of a static RBE is questioned by experimental findings and in silico simulations with ongoing efforts to elaborate on variable RBEs, the role of LET, and the underlying biological background [2–6]. Appropriate RBE definition is further complicated by the multitude of variables involved, such as endpoint, tissue, or experimental model investigated. Needless to say, a broadened knowledge on differently balanced cellular processes subsequent to proton irradiation in comparison to photon irradiation could supply radiation oncologists with a novel rationale for combinatorial approaches with RBE-modulating agents.

A physical characteristics of proton radiotherapy is the increase in LET towards the distal end of the SOBP. Irradiation with an elevated LET induces more complex respectively clustered DNA lesions than yielded by conventional low LET photon irradiation [7–11]. These differences in the pattern of DNA double strand breaks (DSB) bear the potential to induce different cellular pathways and/or require different DNA repair processes.

The two major pathways involved in DNA DSB repair are non-homologous end joining (NHEJ) and homologous recombination repair (HRR). In brief, NHEJ can be summarized as a fast process, responsible for the vast number of repaired DSBs upon ionizing irradiation, and is present throughout the cell cycle [12, 13]. NHEJ involves minor DNA end processing and as a result comprises the risk of accidental mutations prior to relegation of the strands. HRR on the other hand is limited to the presence of duplicated DNA during S- and G2 phase, involves extensive single-strand resection, Rad51-mediated strand invasion for recombination, DNA polymerization, and the resolution of the resulting complex DNA structure. As a result, HRR is generally slower and consumes more cellular resources but is ultimately less error-prone than NHEJ. Recapitulating, the cellular response to radiation-induced DSBs is a fine-tuned balance of repair mechanisms, depends on cell cycle phase, and is essential to maintain genome integrity [13–15].

The particular importance of HRR in response to proton irradiation was demonstrated by diverse approaches. HRR deficiency in Chinese hamster ovary (CHO) cells resulted in a proton irradiation-sensitive phenotype as indicated by increased RBEs [16]. A similar hypersensitivity towards proton irradiation was detected in HRR-corrupted human cancer cell lines and could be replicated by siRAD51 treatment [17] and the broad-range histone deacetylase inhibitor SAHA (Vorinostat) [18], which eventually downregulates Rad51 protein levels. These findings were further corroborated by a screening of lung cancer cell lines indicating a correlation of HRR deficiency with an increased RBE and a decreased RBE in cells with elevated levels of Rad51 mRNA and protein [19]. More focused research on deficiencies in HRR key proteins such as Slx4 and Mus81 [20] equally suggested an increased importance of HRR in response to proton compared to conventional photon irradiation. Consequently, interference with HRR constitutes a promising target to sensitize cancer cells for proton radiotherapy.

Pharmaceutical modulation of HRR is a clinically difficult target and administration of many inhibitors of HRR is limited to in vitro experiments. This limitation is based on the difficulty of providing pharmaceutically unfeasible drug concentrations or due to severe side effects provoked in the patient. A possible alternative is provided by the small molecule, HSP90-inhibiting ganetespib which downregulates versatile DSB repair proteins including Rad51 and BRCA2 [21–24] and eventually suppresses HRR. Furthermore, the elevated intrinsic proteotoxic stress in tumor cells upregulates the activated, high-affinity form of chaperone HSP90 and hence provides a certain tumor-specific vulnerability [25]. In addition, ganetespib demonstrated a good tissue penetrance and pharmaceutical profile [26, 27] and was administered in numerous clinical trials [28–30]. However, current radiobiology-oriented research is limited to photon and high-LET carbon ion irradiation in combinations with HSP90 inhibitors [31–34]. Here we investigated the radiosensitizing effect of the HSP90 inhibitor ganetespib in combination with reference photon and proton irradiation of two different positions along the SOBP, having different LET values. Ganetespib administration and proton radiotherapy were designed to replicate the clinical situation as close as experimentally feasible to reduce undesirable uncertainties and foster a prospective clinical application.

## Methods

### Cell culture

A549 and FaDu cells were maintained in RPMI 1640 (2 mM Lglutamine, 10% FBS, 1 mM sodium pyruvate, 25 mM HEPES, 100U/ml penicillin, 100 streptomycin, and 0.25 µg/ml amphotericin B) at regular cell culture conditions (37 °C, 5% CO_2_, humidified atmosphere) and subcultured prior to reaching confluency. Cell pellets were sent in for cell line authentication (Eurofins) by STR profiling (Applied Biosystems™ AMPFLSTR™ Identifiler™ Plus) and compared using CLASTR 1.4.4. Cells were regularly tested mycoplasma-free by a commercial kit (Lonza, MycoAlert) and during microscopy with DAPI (5 μg/ml in PBS) as DNA-intercalating dye.

For drug treatment, ganetespib was dissolved and diluted in DMSO to achieve a stock concentration of 10 μM and stored at − 20 °C. For treatment, fresh supplemented medium was mixed with ganetespib to achieve the defined concentration (c_DMSO_ ≤ 0.1%) and used to replace the previous growth medium approximately 1 h before irradiation. For sham treatment, the same amount of DMSO was added to the medium and exchanged similarly to exclude undesired and unnoted effects by DMSO itself. Cells were retained in drug/sham-containing medium until fixation/harvesting.

For irradiation, exponentially growing cells were seeded in 9 cm^2^ slide flasks at a cell density of 5.000–15.000 cells/cm^2^ and at least 36 h prior to irradiation to avoid cell cycle synchronisation. For horizontal irradiation and to eliminate proton beam inaccuracies, the flasks had to be turned upwards and were consequently filled air-bubble free with the respective cell culture medium. After irradiation, the medium was completely removed and replaced with fresh drug/DMSO-containing medium to restore gas exchange.

### Photon irradiation

For reference irradiation, 200 kV photons were administered using a horizontal irradiation cabinet (YXLON, TU32-D03, 20 mA, 5.5FOC, filtration: 3 mm Be + 3 mm Al + 0.5 mm Cu). Slide flasks where positioned at 40 cm distance from the beam exit window in a PMMA holder to provide a homogeneous distribution and a dose rate of 1.28 Gy/min.

### Proton irradiation

Slide flasks were submerged in a water/PMMA phantom at a proximal (55 mm depth, LET 2.1 keV/µm) and a distal position (105 mm depth, LET 4.5 keV/µm) of a SOBP (shown in Additional file 1: Figure S1). The SOBP ranged from 40 to 120 mm depth which correlates to proton beam energies ranging from 66.5 to 136 MeV. Dose-averaged LETs are obtained from Monte Carlo simulations by the treatment planning software (RayStation). Protons were administered by spot scanning with dosimetric characterization and treatment plans as described in a previous publication [35].

### Proliferation assay

For pilot dose finding, we seeded 500 cells in 96-well plates, waited for 2 h to allow cell adherence, and administered varying concentrations of ganetespib or sham. Additionally, plates were sham irradiated (0 Gy) or received 3 Gy or 6 Gy of photon irradiation. Viability was determined by incubation with alamarBlue for 4 h and photometric determination of the occurring color change at multiple time points in triplicates. For plotting and statistical comparison, we utilized GraphPad Prism (Version 9.2.0) and performed unpaired, two-tailed t-test with Welch’s Correction.

### Clonogenic assay

To determine the reduced clonogenicity after irradiation and/or drug treatment, we seeded exponentially growing cells as described above. Cells were pretreated with ganetespib or sham 1 h prior to irradiation with doses of 0 Gy (sham irradiation), 2 Gy, 4 Gy, and 6 Gy, respectively. Subsequent to irradiation, the cells were detached from the slide flasks, counted, diluted accordingly and plated into 6-well plates in sextuplicate to achieve approximately 20–300 surviving clones per well. After 7 (A549) respectively 14 days (FaDu), the cells were washed with PBS, fixed with MeOH:HAc (3:1), air-dried and subsequently stained with crystal violet (2%). Counting was performed manually in a blinded fashion and clones above 50 cells considered as a surviving cell.

### Linear-quadratic model, formulas, and statistics

The plating efficiency was derived from unirradiated samples and considered when calculating the fraction of surviving clones. The clonogenic assays were performed in triplicate and a linear-quadratic model applied to obtain α and β and calculate DMF and RBE at 50%, 25%, and 10% survival rate, respectively. Following formulas were applied:

$${\text{Linear-quadratic}}\;{\text{model}}\quad {\text{y}} = \exp ( - (\upalpha *{\text{x}} +\upbeta *{\text{x}}^{2} )$$

y surviving fraction, x dose [Gray], weighted by 1/y^2^ (minimizing relative squares).

Dose-modifying factor

$${\text{DMF}}_{{\text{y}}} = \frac{{\text{dose}}_{{{\text{y survival}},{\text{ sham}}}}} {{\text{ dose}}_{{{\text{y survival}},{\text{ ganetespib}} - {\text{treated}}}}}$$

Relative biological effectiveness

$$ {\text{RBE}}_{{\text{y}}} = \frac{{\text{dose}}_{{{\text{y survival}},{\text{ proton}},{\text{ sham}}}}} {{\text{ dose}}_{{{\text{y survival}},{\text{ photon}},{\text{ sham}}}}} $$

$${\text{respectively}} = \frac{{\text{dose}}_{{{\text{y survival}},{\text{ proton}},{\text{ ganetespib}} - {\text{treated}}}}} {{\text{ dose}}_{{{\text{y survival}},{\text{ photon}},{\text{ ganetespib}} - {\text{treated}}}}} $$

For statistical comparison, DMF and RBE values were plotted in GraphPad Prism (Version 5.03) and unpaired, two-tailed *t* test with Welch’s Correction performed.

### Cell cycle analysis

Cells were seeded, treated and irradiated with a dose of 4 Gy as described above. Cells were harvested at 0 h, 8 h, 24 h, and 48 h to investigate the initial cell cycle distribution as well as irradiation-induced cell cycle alterations. Consequently, medium was replaced with BrdU-containing (10 µM) medium 1 h before fixation to label DNA-polymerizing cells. At the respective time points, cells were trypsinised, and fixated by addition of ice-cold EtOH (80%) while vortexing. Fixed cells were stored at − 20 °C until all samples were collected and available for staining.

To stain the cells, they were treated with 4 M HCl (10 min) to denature DNA, neutralized with PBS, incubated in blocking buffer (BSA 0.5%, Tween 20 0.1%, in 1 × PBS) for 1 h at room temperature and subsequently in staining solution (5 μg/ml antiBrdU-FITC, 5 μg/ml propidium iodide, 80 μg/ml RNAse A, in blocking buffer) for 1.5 h, in the dark, and at room temperature. Stained cells were washed with PBS, filtered through a cell strainer before flow cytometry (FACSCanto II, BD Biosciences) and the obtained data analyzed with FlowJo (Version 10.5.2). We gated for cells in G1 phase (PI 2N & BrdU neg.), S & early G2 phase (PI 2N-4N & BrdU pos.), and late G2 & M-phase (PI 4N & BrdU neg.).

We were able to obtain a duplicate of A549 samples which was seeded, irradiated, and stained independently. A third iteration or repetition with FaDu cells could not be obtained due to beam time limitations and transport constraints.

### Immunoblotting

A549 cells were handled similarly to the flow cytometry samples and seeded, treated, and irradiated with a dose of 4 Gy as described above. Upon harvest at 8 h and 24 h post-irradiation, cells were washed with PBS, lysis buffer (2% SDS, 10% glycerin, 50 mM Tris, 6.8 pH) added, and homogenously distributed with a cell scraper. The obtained lysates were heated for 95 °C (10 min) and stored at − 20 °C. Similar cell numbers were seeded and because of the low protein concentrations determined in pilot experiments, no dilution step was necessary.

For SDS-PAGE, a separation gel (10% acrylamide, 1.5 mm thick, pH 8.8) with stacking gel (pH 6.8) on top was self-cast and a gel electrophoresis system (BioRad, Mini-PROTEAN) utilized. Samples were mixed with 5 × sample buffer (5% SDS, 25% glycerin, 0.25% bromophenol blue, 125 mM Tris, 10% freshly added 2-mercaptoethanol, pH 6.8) and 30 μl of this mixture loaded per gel slot.

The proteins were electrophoretically separated (80 V until front reaches separation gel, 100 V until front reaches end of separation gel), blotted onto a PVDF membrane (70 min, 80 V), the membrane blocked for 1 h with blocking buffer (5% non-fat dry milk in TBS-T) and incubated overnight at 4 °C with primary antibody diluted in blocking buffer. Membranes were washed three times for 10 min each with TBS-T (8 g/l NaCl, 0.2 g/l KCl, 3 g/l Tris, 0.1% Tween, pH 7.4), incubated with secondary HRP-labelled antibody (1 h, room temperature), and subsequently washed again three times for 10 min each with TBS-T. Protein detection was accomplished by overlaying the membrane with HRP substrate and imaging chemiluminescence with a photo cabinet. The visualized bands were quantified by Fiji (Version 1.52 g) and results normalized to the β-actin loading control. An untreated control sample was included in each run in the first lane and utilized to compare plotting efficiency and protein levels in between membranes.

### Immunofluorescence nuclear γH2AX foci staining

A549 cells were seeded, treated and irradiated with a dose of 2 Gy as described above. 0.5 h and 24 h after irradiation, cells were fixed with formaldehyde-based fixative (4%, 15 min, 4 °C) and slide flasks filled up with 1 × PBS for transport and storage at 4 °C.

For staining, the upper chamber part was carefully removed and the fixed cells on the plastic microscopy slide made accessible for following staining steps. Homogenous antibody and dye distribution was assured by careful overlay with parafilm. Cells were incubated with blocking buffer (10% FBS, 0.2% Triton X-100, in 1 × PBS) for 1 h at room temperature followed by staining solution (1:50 anti-γH2AX-AF647, in blocking buffer) overnight at 4 °C in the dark, and subsequently washed three times with blocking buffer. Slides were subsequently stained with DAPI (5 µg/ml, in 1 × PBS) for 30 min at room temperature in the dark, before washing three times in PBS. Anti-fade mounting medium and a cover slip were carefully overlayed and the edges sealed with nail polish for storage at 4 °C in the dark.

Images were recorded with an immunofluorescence microscope (> 50 cells imaged per condition, Leica Thunder). Foci numbers were assessed by applying the “find maxima”-function of open source Fiji (Version 1.52 g) and the data plotted in GraphPad Prism (Version 5.03).

### Antibodies

For cell cycle analysis we used antiBrdU-FITC (mouse, Roche, 11202693001). We further utilized antibodies targeting Rad51 (rabbit, BioAcademia, 70-002) and β-actin (mouse, SigmaAldrich, A5441-0.2 ml) for immunoblotting as well as HRP-labelled secondary antibodies against rabbit (mouse, Santa Cruz Biotechnology, sc-2357) and against mouse (sheep, GE Healthcare, NA931V) for chemiluminescent detection. For the staining of nuclear DNA repair foci we employed an antibody targeting p.S139 H2AX (mouse, BD Pharmingen, 560447) labelled with AF647.

## Results

### Pilot dose finding studies

**Fig. 1:**
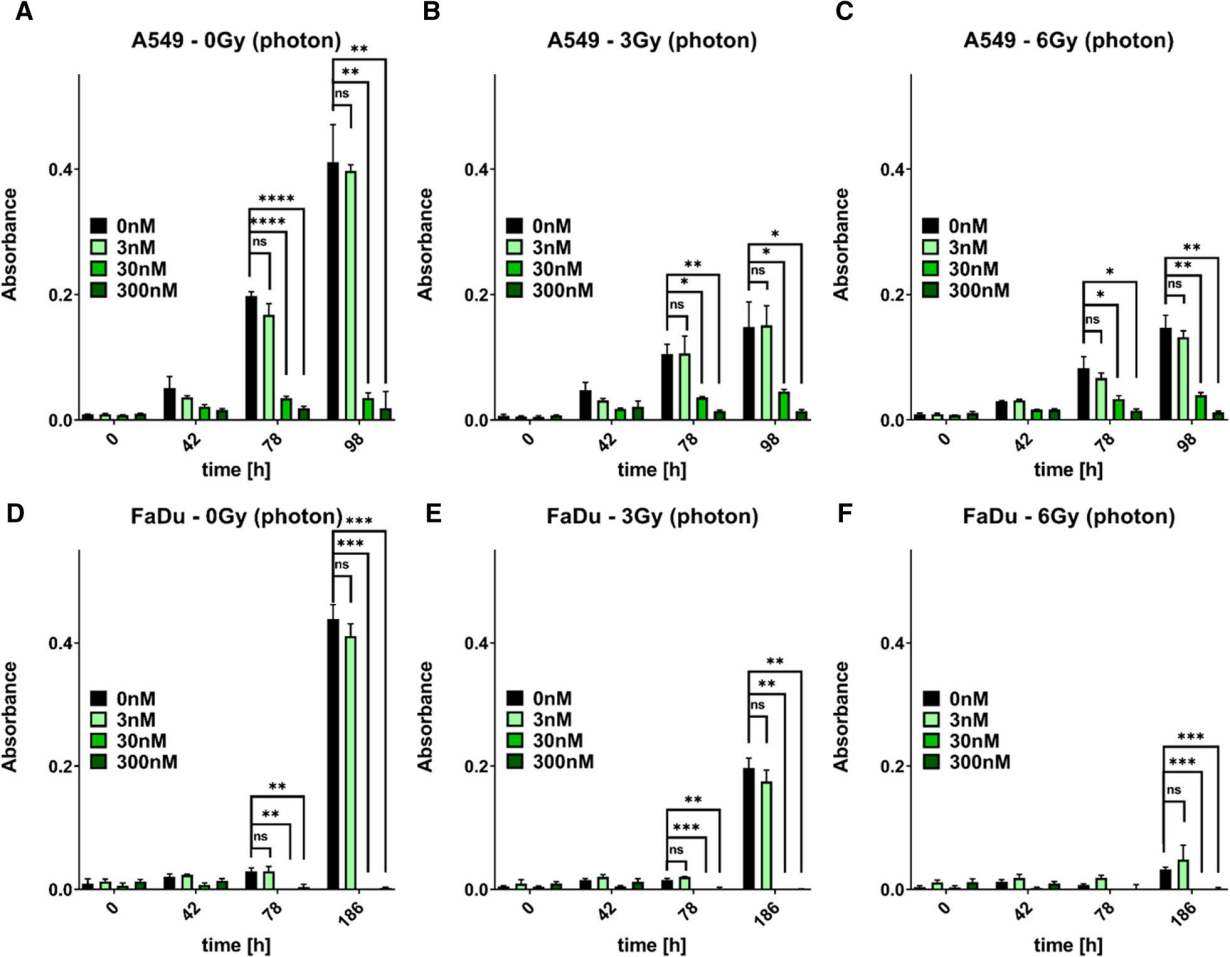
Proliferation assay of A549 and FaDu cells, treated with varying concentrations of ganetespib and irradiated with sham, 3 Gy, or 6 Gy of reference photon irradiation. Proliferative capacity was determined by alamarBlue assay of A549 (**A**–**C**) and FaDu (**D**–**F**) cells. Cells were irradiated with 0 Gy (**A**, **D**), 3 Gy (**B**, **E**), or 6 Gy (**C**, **F**) of reference photon irradiation. 30 nM and 300 nM ganetespib are sufficient to inhibit proliferation of A549 and FaDu cells irrespective of the administration of sham or actual irradiation. Importantly, there is no significant difference between 0 and 3 nM ganetespib, irrespective of the photon radiation dose administered. Statistical testing was performed by unpaired, two-tailed *t* test with Welch’s Correction with error bars indicating the standard deviation

We performed proliferation assays to establish a sublethal dose of ganetespib because of its shorter turnaround time. Both, A549 and FaDu cells, demonstrated a clear growth inhibition by 30 nM and 300 nM ganetespib (Fig. 1). As expected, 3 Gy and 6 Gy reference photon irradiation also caused a reduction in viability compared to sham (0 Gy) irradiated cells. Importantly, low-dosed ganetespib did not cause a significant viability difference when comparing cells treated with 0 nM or 3 nM ganetespib, neither without irradiation nor in combination with 3 Gy or 6 Gy of reference photon irradiation.

**Fig. 2:**
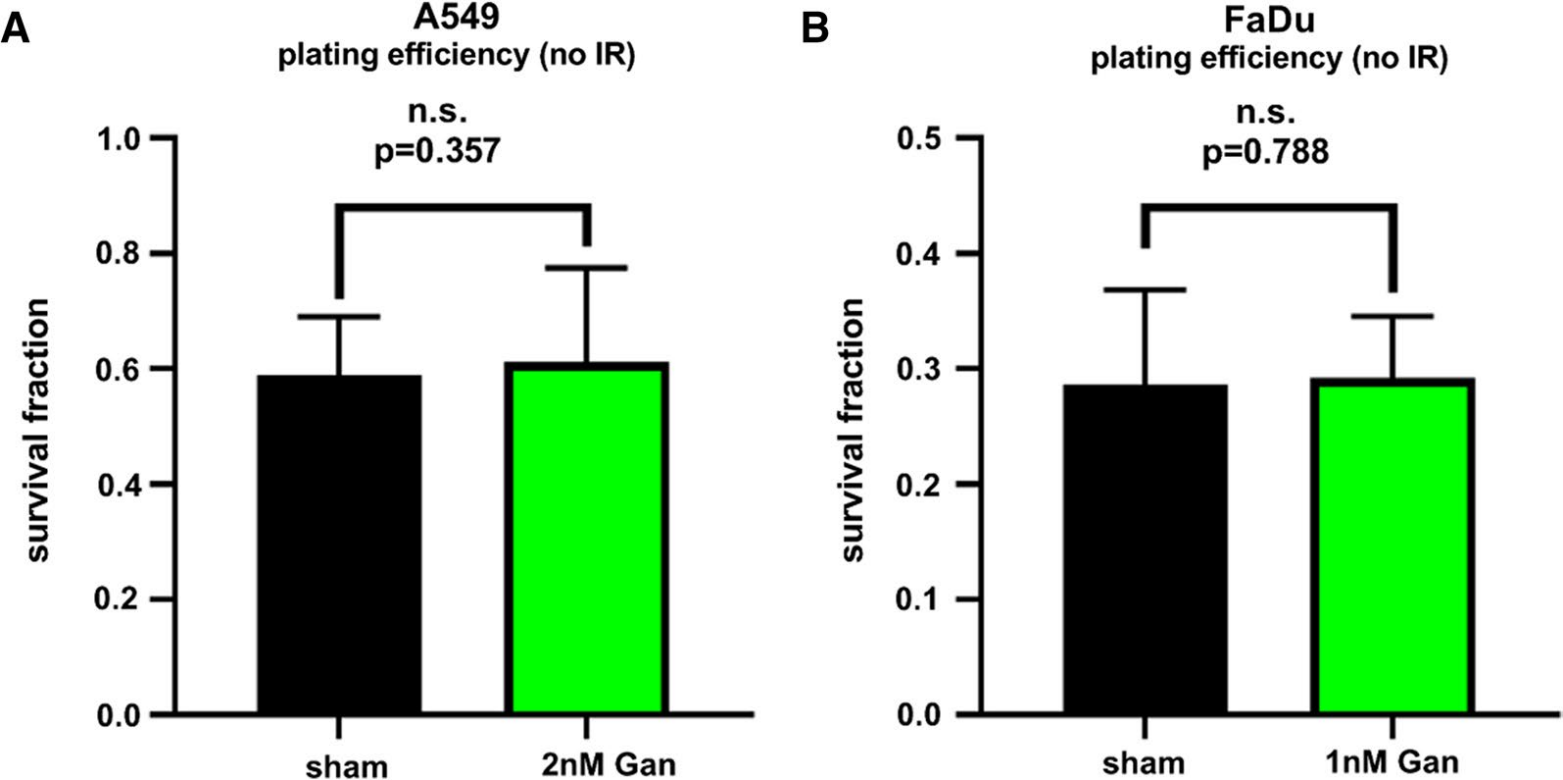
Plating efficiency of unirradiated A549 and FaDu cells is not affected by the administered low-dosed ganetespib. Clonogenic cell survival of **A** A549 cells and **B** FaDu cells was investigated after sham treatment or the administration of 2 nM or 1 nM Ganetespib, respectively. The reduced dose compared to previous data from proliferation assay accounted for the additional stress induced by the drug treatment preceding the plating procedure. Importantly, no difference in plating efficiency was observed at the investigated reduced dose. Statistical testing was performed by unpaired, two-tailed *t* test with Welch’s Correction with error bars indicating the standard deviation

While the initial proliferation assays were performed by treatment of pre-plated cells, we had to switch these steps to the plating of pre-treated cells for our clonogenic cell survival assays. This procedural difference became necessary due to the irradiation set-up of the horizontal proton beam line utilized. Consequently, we aimed to avoid a significant difference in plating efficiency and cellular fitness by ganetespib itself in order focus on the combinatorial effects of ganetespib with photon and SOBP proton irradiation, respectively. Consequently, we lowered our initial dose range of 3 nM Ganetespib as determined by the proliferation assay, to 2 nM for A549 cells and 1 nM for FaDu cells for the clonogenic cell survival assays, respectively. This reduction accounted for the additional stress induced by ganetespib administration in advance of plating. No significant difference in plating efficiency between sham and ganetespib-treated cells could be observed at this reduced ganetespib concentration (Fig. 2).

### Ganetespib sensitizes A549 cancer cells for proton radiotherapy

**Fig. 3:**
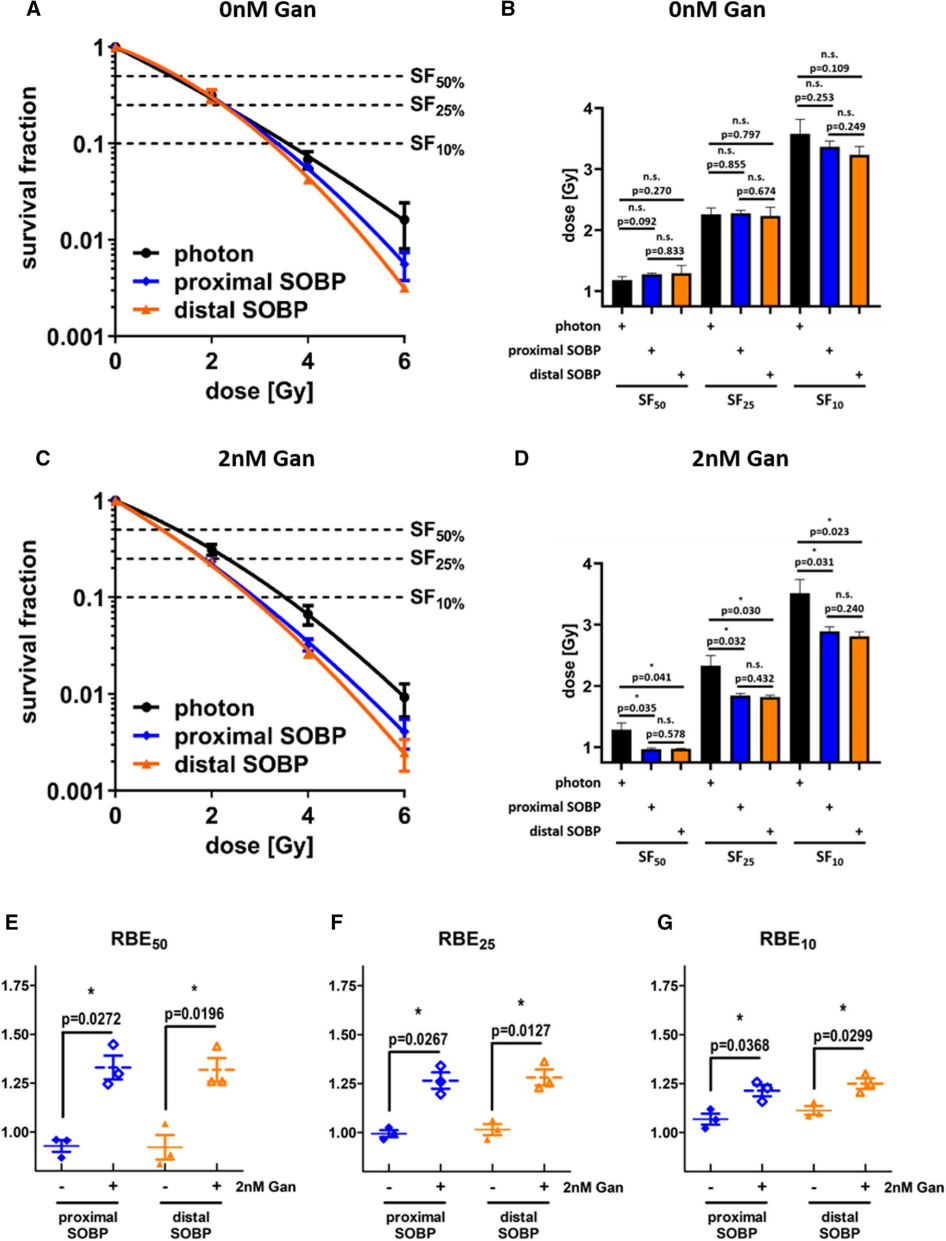
Clonogenic cell survival of A549. Clonogenic cell survival of sham treated A549 cells irradiated with each of the investigated radiation modalities (**A**) and the corresponding dose to achieve SF_50_, SF_25_, and SF_10_ (**B**). Importantly, no statistical significant difference can be observed in sham treated A549 cells. Similar clonogenic data obtained in A549 cells treated with 2 nM ganetespib (**C**) highlights a proton-exclusive radiosensitizing effect. This sensitization by 2 nM ganetespib significantly reduces the dose required to reach SF_50_, SF_25_, and SF_10_ in A549 cells irradiated with proximal and distal SOBP protons compared to reference photon IR (**D**). Consequently, the RBE of both proton modalities is significantly higher by 10–40% in A549 cells upon ganetespib treatment compared to sham treatment (**E**–**G**). Proximal and distal SOBP corresponds to a LET of 2.1 respectively 4.5 keV/μm and statistical testing was performed by unpaired, two-tailed t-test with Welch’s Correction with error bars indicating the standard deviation

We irradiated sham treated A549 cells with all discussed irradiation modalities (shown in Fig. 3A) prior to platting them at defined cell numbers. The dose corresponding to 50% (SF_50_), 25% (SF_25_), and 10% (SF_10_) cell survival was obtained by applying the linear-quadratic model to the clonogenic survival data (shown in Fig. 3B). Interestingly, there is no statistically significant difference in between photon versus proximal SOBP, photon versus distal SOBP, or proximal SOBP versus distal SOBP irradiation in sham-treated A549 cells at the examined survival fractions. In contrast, treatment with 2 nM ganetespib sensitized A549 cells for both proximal and distal SOBP proton irradiation (Fig. 3C) in comparison to reference photon irradiation. As a result, A549 cells treated with 2 nM ganetespib reached SF_50_, SF_25_, and SF_10_ at statistically lower doses when combined with proximal and distal SOBP proton IR than when administered with reference photon IR (Fig. 3D). Importantly, this difference also becomes evident when converting to RBE (Fig. 3E–G). At all investigated survival fractions, the RBE of proximal and distal SOBP proton IR (both in reference to photon IR) was significantly higher in ganetespib-treated A549 cells compared to sham treated cells, with RBE increases ranging from 10 to 40%.

**Fig. 4:**
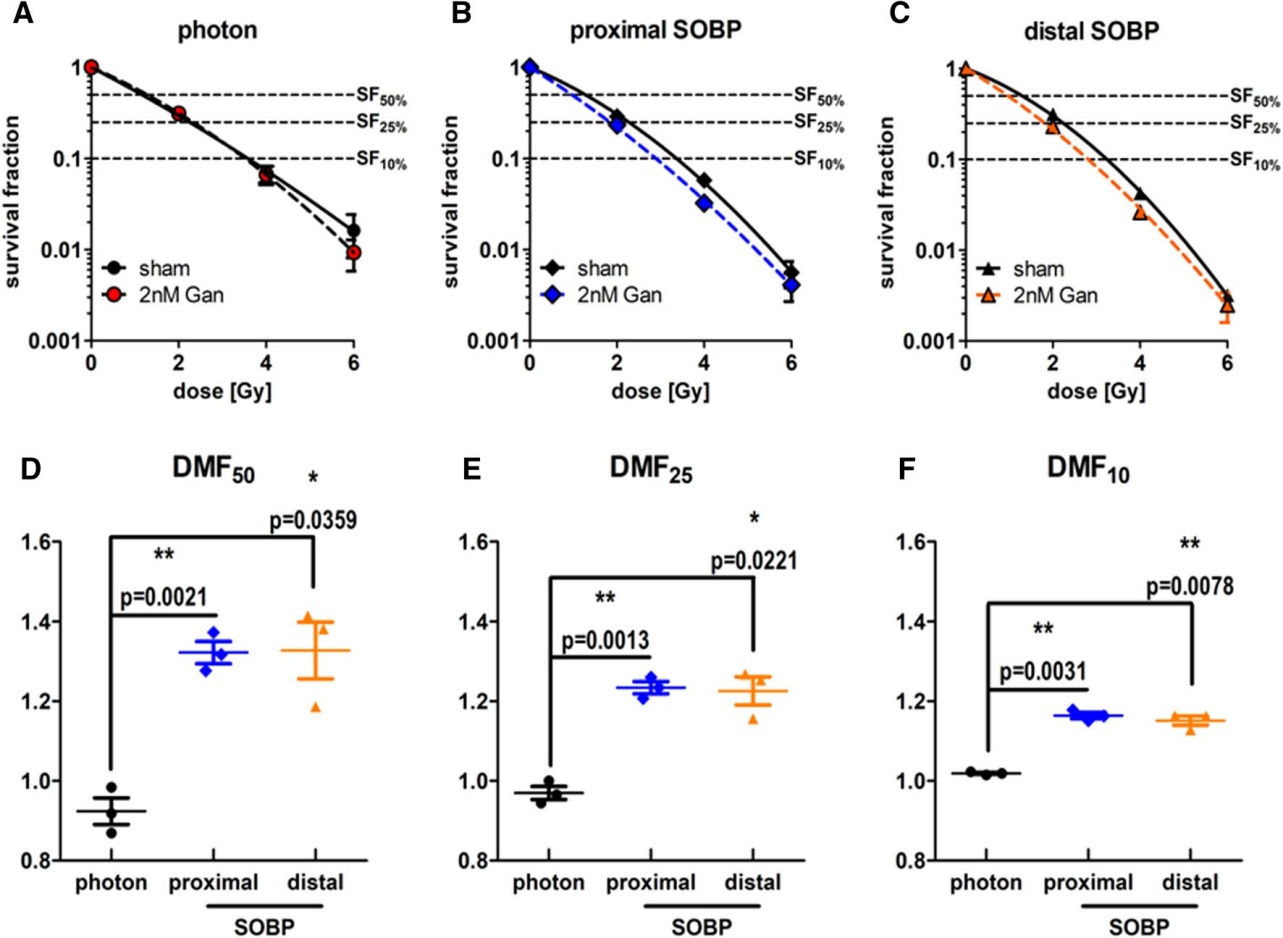
Dose-modifying factor of ganetespib in A549 cells in response to photon, proximal SOBP, and distal SOBP irradiation. A549 cells irradiated with photon reference IR do not exhibit a radiosensitizing effect upon ganetespib treatment (**A**). In contrast, the linear-quadratic model highlights differences in A549 cells upon irradiation with **B** proximal SOBP and **C** distal SOBP protons in dependence of sham or ganetespib treatment. Consequently, the DMF of ganetespib at **D** 50%, **E** 25%, and **F** 10% cell survival is significantly higher in A549 cells irradiated with either of the two proton modalities compared to reference photon IR. Proximal and distal SOBP corresponds to a LET of 2.1 respectively 4.5 keV/μm and statistical testing was performed by unpaired, two-tailed *t* test with Welch’s Correction with error bars indicating the standard deviation

When focusing on the DMF of ganetespib, similar findings can be observed (Fig. 4A–C). The DMF of ganetespib in A549 cells irradiated with reference photons is close to 1 at all investigated survival fractions (Fig. 4D–F), suggesting only negligible radiosensitization for reference photon irradiation. In contrast, the DMFs of ganetespib in proximal and distal SOBP proton-irradiated A549 cells was significantly increased (1.15–1.33) at all investigated survival fractions, emphasizing a sensitization for proton irradiation by low-dosed ganetespib.

Additional tabular information on α, β, DMF, and RBE for A549 cells can be found in the supplements (shown in Additional file 1: Tables S1 and S2).

### Ganetespib sensitizes FaDu cancer cells for proton radiotherapy

**Fig. 5:**
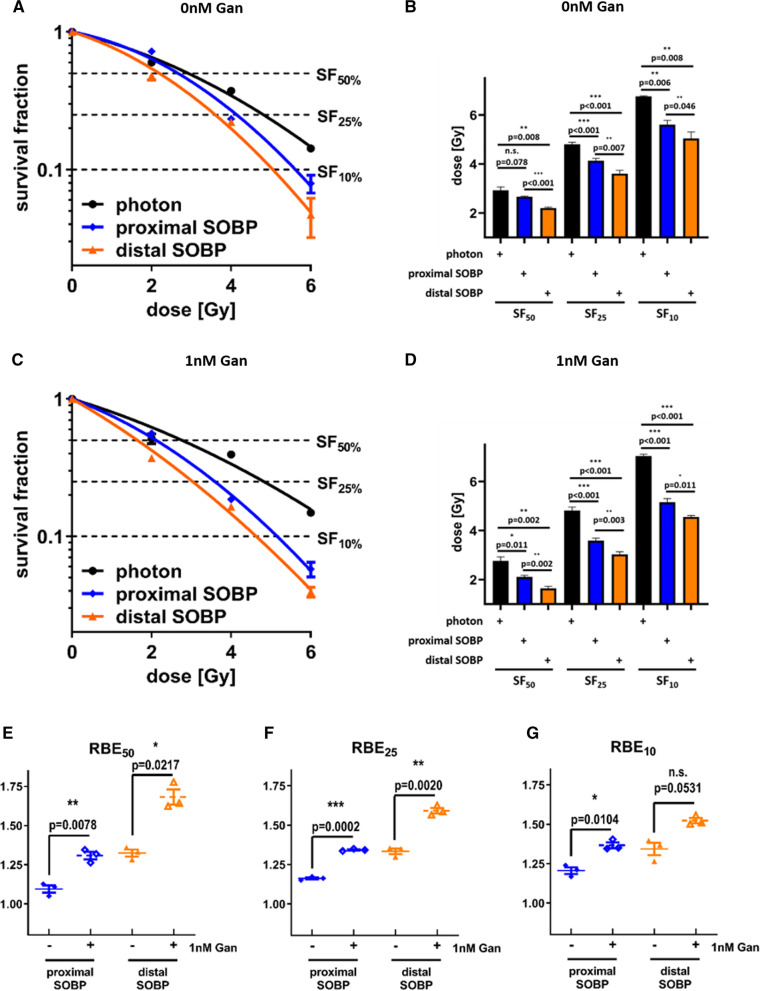
Clonogenic cell survival of FaDu. Clonogenic cell survival of sham treated FaDu cells irradiated with each of the investigated radiation modalities (**A**) and the corresponding dose to achieve SF_50_, SF_25_, and SF_10_ (**B**). Interestingly, sham treated FaDu cells exhibit significant differences in radiosensitivity increasing from photon to proximal SOBP to distal SOBP proton IR. Nonetheless, 1 nM ganetespib further increased this sensitivity for SOBP proton irradiation (**C**) highlighting a proton-exclusive radiosensitizing effect. This sensitization by 1 nM ganetespib significantly reduces the dose required to reach SF_50_, SF_25_, and SF_10_ in FaDu cells irradiated with proximal and distal SOBP protons compared to reference photon IR (**D**). Consequently, the RBE of both proton modalities is significantly higher by 10–30% in FaDu cells upon ganetespib treatment compared to sham treatment at all examined SFs except for distal SOBP proton irradiation at SF_10_ (**E**–**G**). Proximal and distal SOBP corresponds to a LET of 2.1 respectively 4.5 keV/μm and statistical testing was performed by unpaired, two-tailed *t* test with Welch’s Correction with error bars indicating the standard deviation

Parallel experiments were performed with the HNSCC cell line FaDu which was irradiated with all discussed irradiation modalities prior to platting them at defined cell numbers (Fig. 5A). In comparison to A549 cells, FaDu cells demonstrated a significant difference when comparing the dose necessary to achieve SF_50_, SF_25_, and SF_10_, reducing from photon IR to proximal SOBP to distal SOBP proton IR (Fig. 5B). Nonetheless, ganetespib administration equally sensitized FaDu cells for proximal and distal SOBP proton irradiation while not altering the response to photon IR (Fig. 5C), corroborating our observations with A549 cells. As a result, the higher sensitivity of FaDu cells for proton irradiation increased upon ganetespib treatment even further, following the same order as described above and retaining statistical significance (Fig. 5D). Importantly, these differences also manifested upon calculation of RBE (Fig. 5E–G) for all investigated survival fractions. In detail, the RBE of proximal and distal SOBP proton IR compared to reference photon IR increased in FaDu cells by 10–30% upon treatment with 1 nM ganetespib and reached significance for all examined survival fractions except for distal SOBP proton irradiation at SF_10_.

**Fig. 6:**
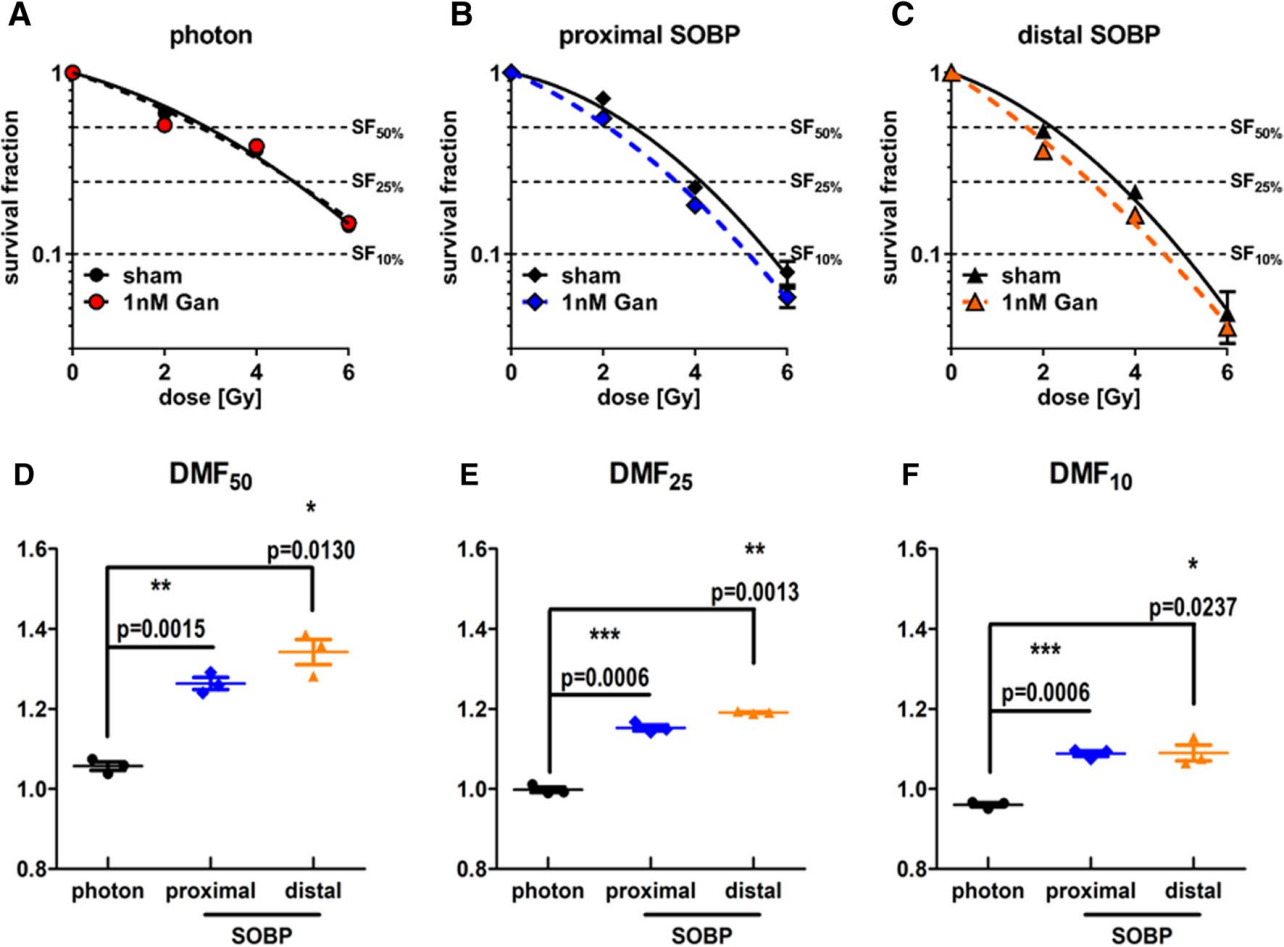
Dose-modifying factor of ganetespib in FaDu cells in response to photon, proximal SOBP, and distal SOBP irradiation. FaDu cells irradiated with photon reference IR do not exhibit a radiosensitizing effect upon ganetespib treatment (**A**). In contrast, the linear-quadratic model highlights differences in FaDu cells upon irradiation with **B** proximal SOBP and **C** distal SOBP protons in dependence of sham or ganetespib treatment. Consequently, the DMF of ganetespib at **D** 50%, **E** 25%, and **F** 10% cell survival is significantly higher in FaDu cells irradiated with either of the two proton modalities compared to reference photon IR. Proximal and distal SOBP corresponds to a LET of 2.1 respectively 4.5 keV/μm and statistical testing was performed by unpaired, two-tailed *t* test with Welch’s Correction with error bars indicating the standard deviation

When focusing on the DMF of ganetespib, similar findings can be observed (Fig. 6A–C). The DMF of ganetespib in FaDu cells irradiated with reference photons is approximating 1 at all investigated survival fractions (Fig. 6D–F), suggesting only negligible radiosensitization for reference photon irradiation. In contrast, the DMFs of ganetespib in proximal and distal SOBP proton-irradiated FaDu cells was significantly increased (1.09–1.34) at all investigated survival fractions. This emphasizes the sensitizing effect of low-dosed ganetespib exclusively for proton irradiation in FaDu cells and corroborates our results observed in A549 cells.

Additional tabular information on α, β, DMF, and RBE for FaDu cells can be found in the supplements (shown in Additional file 1: Tables S1 and S2).

### Effect of proton, photon, and ganetespib treatment on cell cycle distribution in A549

**Fig. 7:**
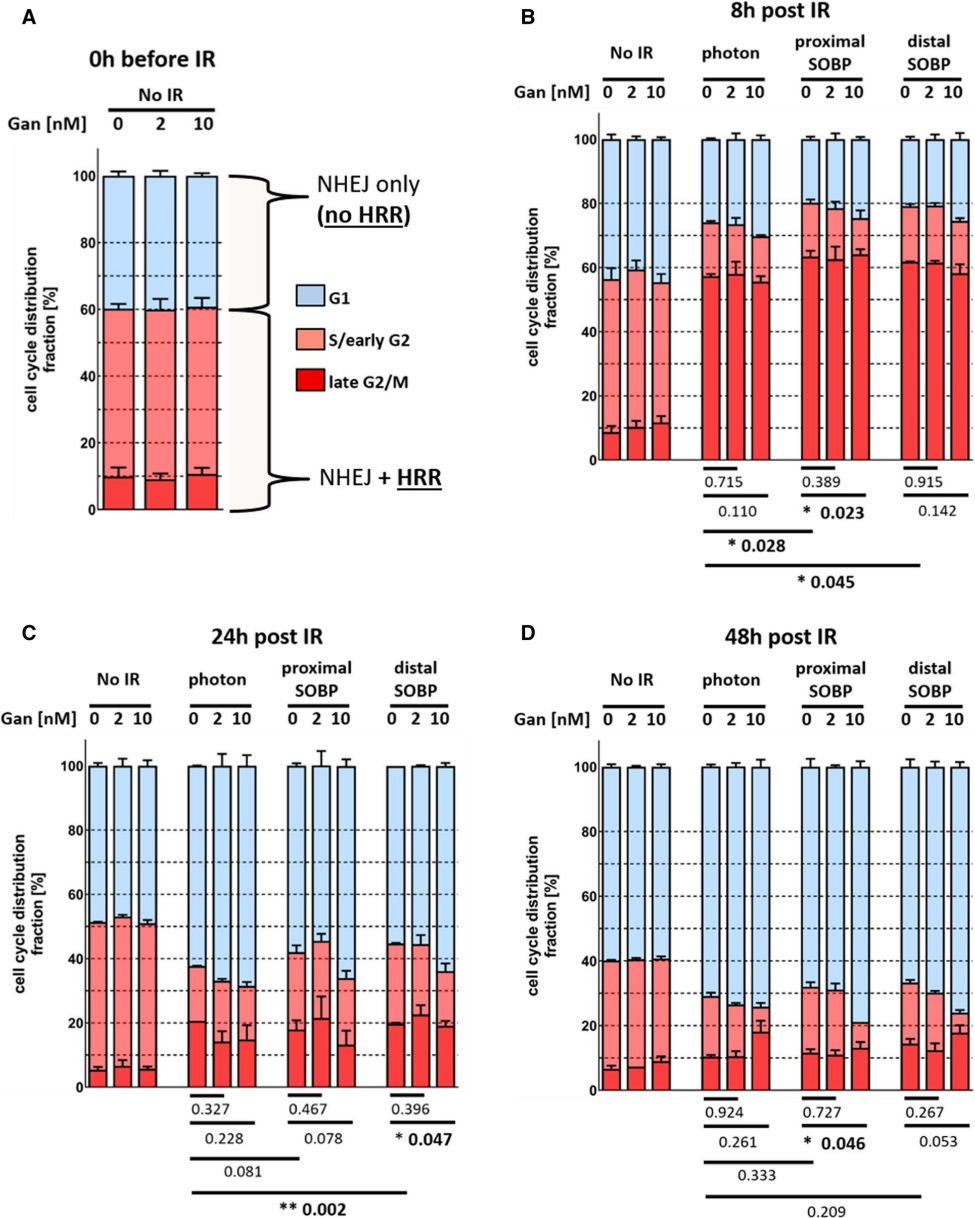
Cell cycle analysis of A549 in response to ganetespib and different radiation modalities. **A** Neither pretreatment nor long term incubation with low-dosed ganetespib altered the fraction of cells in S/G2/M phase (filled red). Cells fixated **B** 8 h, **C** 24 h, and **D** 48 h after receiving 4 Gy of the respective irradiation exhibit an accumulation in S/G2/M phase within 8 h followed by a decline at the 24 h and 48 h time point. A more pronounced fraction of cells in S/G2/M phase could be observed in proton-irradiated cells at the 8 h, 24 h, and 48 h time point which was significant at 8 h for both proton modalities and at 24 h for the distal position. 10 nM ganetespib consistently suppressed the fraction of irradiated cells in S/G2/M phase. Single cells were gated by FSC-H/FSC-A and G1, S and early G2, and late G2 and M phase was gated by DNA content and BrdU uptake. A biological duplicate could be obtained and statistical testing was performed by unpaired, two-tailed *t* test with Welch’s Correction with error bars indicating the standard deviation

To elucidate the initial cellular response to irradiation and explore potential alterations by ganetespib, the cell cycle distribution of A549 was investigated by flow cytometry. The fraction of cells in S/G2/M phase (filled **red** in Fig. 7) is of high interest, since these cells have access to HRR and NHEJ while cells in G1 phase (marked **blue** in Fig. 7) are limited to NHEJ for DNA DSB repair.

Importantly, cell cycle distribution was neither altered by ganetespib pretreatment at the 0 h time point (Fig. 7A) nor at later time points. This excludes cell cycle synchronization as a contributing factor for radiosensitization. The increasing fraction of unirradiated cells in G1 phase over time is most probably caused by the in vitro growth pattern of A549 cells. Despite sufficient space for proliferation at the outer clonal rim, more densely packed central regions were observed microscopically which contributes to an accumulation of spatially growth inhibited cells in G0/G1 phase.

Focusing on sham treated A549 cells, an accumulation of cells in G2 phase was observed 8 h after proton and photon irradiation (Fig. 7B). At the 8 h time point, the fraction of cells in S/G2/M phase was significantly more pronounced in proton- than in photon-irradiated cells by 8.2% (*, 0.03) and 6.8% (*, 0.04) for the proximal and distal SOBP position, respectively. At the 24 h time point, the previous accumulation of cells in G2 phase was observed to shift towards an accumulation in G1 phase (Fig. 7C). Nevertheless, 11.4% (n.s.) and 18.6% (**, 0.002) larger fractions of cells in S/G2/M phase could be observed in response to proximal respectively distal SOBP irradiation compared to reference photon irradiation. At the 48 h time point, most of the irradiated cells accumulated in G1 phase (Fig. 7D). The more pronounced fraction of cells in S/G2/M phase as present at the 24 h time point in proton-irradiated cells was still observed but declined to 10.0% (n.s.) respectively 14.4% (n.s.) for the proximal and distal SOBP position.

10 nM ganetespib consistently decreased the fraction of irradiated cells in S/G2/M phase in comparison to sham-treated (0 nM) cells. The reduction of irradiated cells in S/G2/M phase by 10 nM ganetespib compared to sham treated cells was − 6.0% (n.s.), − 16.5% (n.s.), and − 11,3% (n.s.) for photon-treated cells, − 6.0% (*, 0.02), − 19.3% (n.s.), and − 34.1% (*, 0.05) for the proximal SOBP position, and − 5.8% (n.s.), − 19.2% (*, 0.05), and − 27.9% (n.s.) for the distal SOBP position, at the 8 h, 24 h, and 48 h time points, respectively. The lower dose of 2 nM ganetespib did not demonstrate such a clear trend 8 h, 24 h, and 48 h after irradiation and only marginally altered the fraction of cells in S/G2/M phase.

### Ganetespib downregulates Rad51 protein levels in irradiated A549 cells

**Fig. 8:**
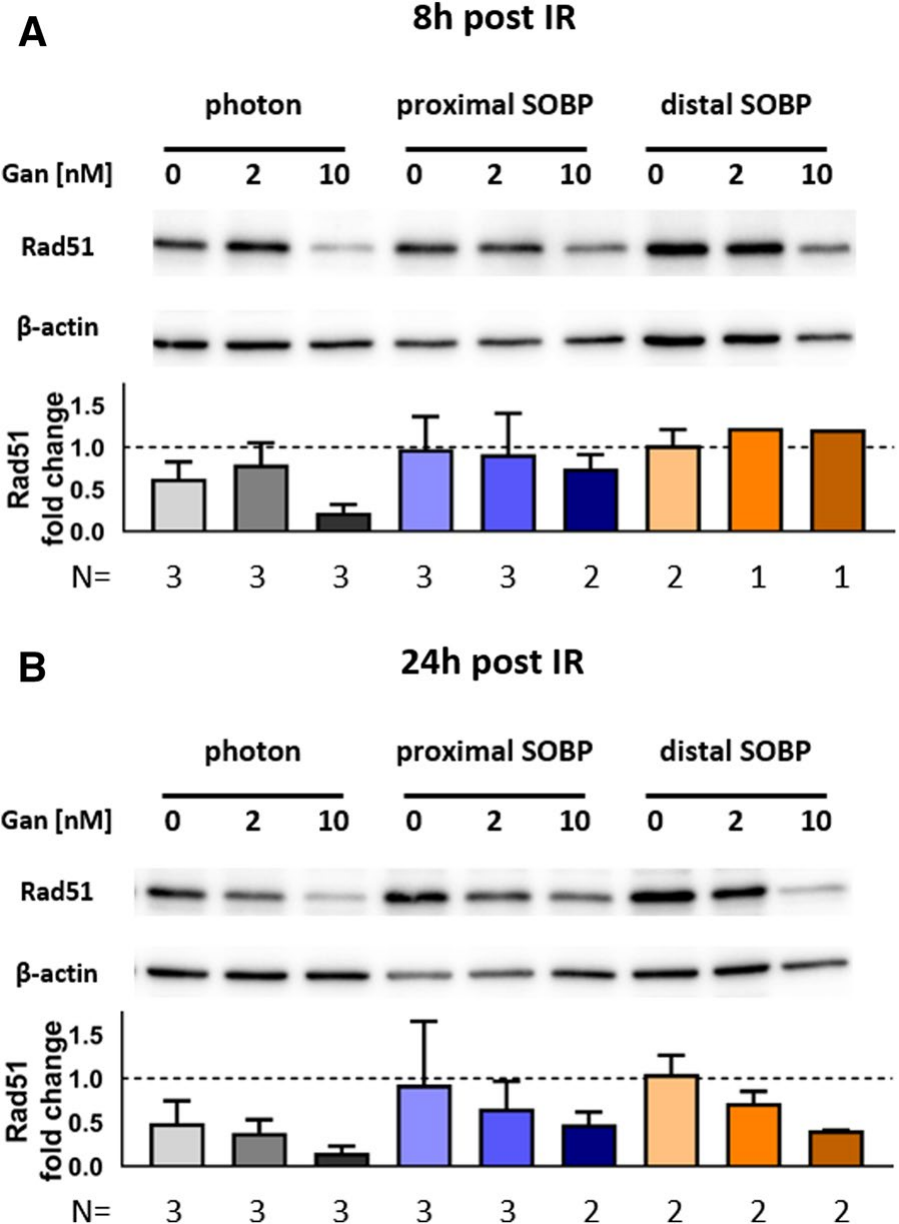
Rad51 immunoblot of A549 cells—8 h and 24 h after irradiation. Immunoblotting of Rad51 protein levels in response to photon irradiation and proton irradiation at a proximal or distal SOBP positions. A549 cells were harvested **A** 8 h and **B** 24 h subsequent to 4 Gy of the indicated type of irradiation. Ganetespib or sham treatment was administered 1 h prior to irradiation and replaced by fresh drug-containing medium after the irradiation. A control lysate was utilized for normalizing protein levels in between the membranes/time point (dotted line) with error bars indicating the standard deviation. Data from 3 independent biological replicates was compiled but differences did not reach significance as assessed by unpaired, two-tailed *t* tests with Welch’s Correction due to the higher variability in immunoblotting results and the limited sample number

We aimed to investigate the downregulation of Rad51, a key protein of HRR, by 2 nM and 10 nM ganetespib as it was published for higher doses. Protein levels of Rad51 in A549 cells were investigated 8 h (Fig. 8A) and 24 h (Fig. 8B) subsequent to irradiation with 4 Gy and concomitant treatment with ganetespib. Importantly, Rad51 proteins levels were downregulated in irradiated cells by 2 nM and 10 nM ganetespib within 24 h upon treatment. This reduction in Rad51 protein levels may be too slow to affect the initial response phase but provides a potential long term mode of action for the radiosensitizing effect of ganetespib. We applied unpaired, two-tailed t-tests with Welch’s Correction but due to the higher variability of immunoblotting data and the limited sample number, we could not reach statistical significance to corroborate the observed trends.

### Effect of proton, photon, and ganetespib treatment on γH2AX repair foci induction and removal

**Fig. 9:**
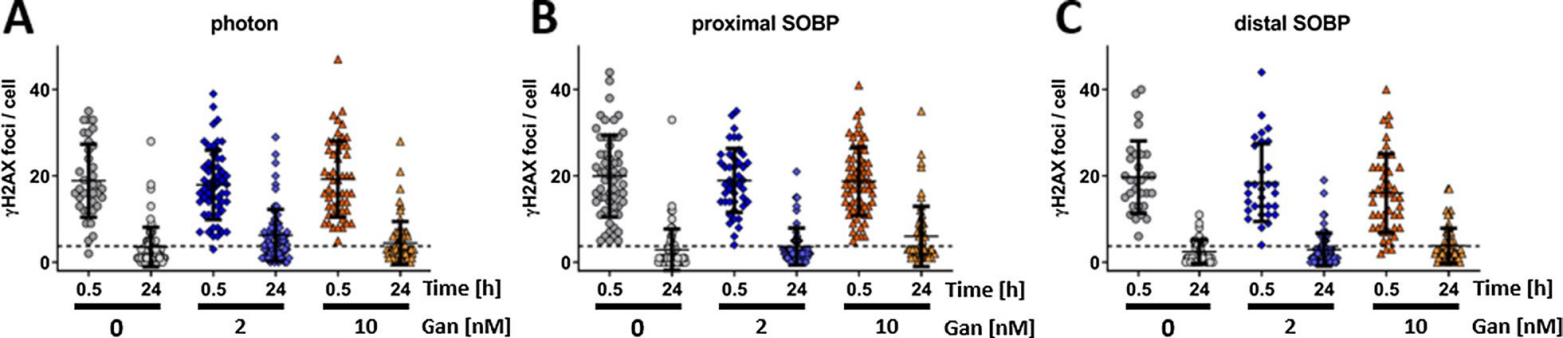
Nuclear γH2AX foci analysis of A549. A549 cells were fixated 0.5 h and 24 h subsequent to irradiation with 2 Gy **A** photons or proton irradiation administered at a **B** proximal and a **C** distal position of a SOBP. The baseline level of γH2AX foci (3.7 foci/cell) in unirradiated A549 cells is illustrated by a dotted threshold line as reference. Ganetespib or sham treatment (0 nM) was administered 1 h prior to irradiation and replaced by fresh drug-containing medium after the irradiation. Nuclear γH2AX foci were stained with fluorophore-labelled antibody and DAPI as nuclear counterstain. Cells were investigated by immunofluorescence microscopy (> 50 cells imaged per condition) and the number of γH2AX foci per nucleus counted with error bars indicating the standard deviation. Unfortunately, we could not obtained biological replicates

DSB repair was investigated in response to a photon beam (Fig. 9A) or at a proximal SOBP (Fig. 9B) and a distal SOBP position (Fig. 9C) in a proton beam. The number of γH2AX foci per cell nucleus were evaluated in unirradiated A549 cells at baseline (3.7 foci/cell, illustrated as dotted threshold line), 0.5 h and 24 h after irradiation with a dose of 2 Gy. These time points are expected to (1) illustrate potential differences in DSB foci induction as well as (2) persisting DSBs which can be expected to interfere with the cell’s health.

A similar number of γH2AX foci were induced independent of the type of irradiation administered. Furthermore, γH2AX foci were removed in a uniform efficiency independent of photon or proton irradiation and reached baseline levels (3.7 foci/cell, dotted threshold line) 24 h after irradiation. Interestingly, ganetespib treatment did neither alter the amount of immediate nor residual γH2AX foci.

## Discussion

This study provides insights into the cellular response to proton irradiation at 2 distinct positions within a SOBP and the combination with HSP90 inhibitor ganetespib. We accomplished this by replicating the clinical irradiation situation as close as experimentally feasible which involved a dosimetrically-defined treatment phantom to mimic relevant tissue depths, the accurate sample positioning at the proximal and distal end of a defined SOBP, as well as advanced spot scanning for proton administration.

We demonstrate for the first time an exclusively proton-sensitizing effect of low-dosed HSP90 inhibitor ganetespib. The radiation dose range applied in this study to obtain RBE and DMF at 50%, 25% and 10% cell survival rate corresponds to the dose range administered during a regular, hypo- or ultrahypofractionated regimen [36]. In compliance with previous research [19], we confirmed the RBE values of sham-treated A549 cells to approximate 1.0, which represents a similar sensitivity for photon and proton radiotherapy. Strikingly, the RBE values of both investigated proton SOBP positions were significantly increased by 10% to 40% in A549 cells upon treatment with 2 nM ganetespib.

Importantly, these promising findings in A549 cells could be reproduced in FaDu cells as second cell line and similar significant RBE increases of 10% to 30% upon ganetespib treatment were observed. The increase of RBE in both cell lines upon ganetespib treatment closely resembles previously described RBE differences in HRR-deficient lung cancer cells compared to HRR-proficient cell lines [19] and as demonstrated by siRNA-mediated knockdown of Rad51 in A549 and CHO cells [16, 17]. Needless to say, both cell lines demonstrated significantly higher DMFs of ganetespib in combination with proximal and distal SOBP proton irradiation than achieved by the combination with reference photon irradiation.

Interestingly, no LET_D_-dependent radiosensitization by ganetespib was evident in either cell line and the RBE increases were similar for the proximal and the distal SOBP position, respectively. Since the investigated SOBP positions cover a narrow LET_D_ spectrum we cannot extrapolate conclusions on LET effects at more elevated values.

Such a LET dependency was suggested and translates to an increasing relative role of HRR upon an increasing LET [37]. In detail, the highest investigated LET in this referenced study was 7.3 keV/μm which resembles a LET as achieved in the more distal fall off region of a SOBP. In contrast, the LET_D_ values investigated in this study are 2.1 and 4.5 keV/μm and in consequence potentially too low and too less apart to observe a clear trend between LET_D_ and radiosensitization by ganetespib. Nonetheless, chosen LET_D_ values closely replicate the clinical situation with the gross tumor volume being covered by a SOBP and higher LETs being frequently limited to the rim of the distal safety margin. Thus, our experimental design more closely resembles the less extreme LET situation in the tumor volume rather than addressing the more distal fall-off region.

We were able to obtain a biological duplicate to investigate cell cycle distribution in A549 cells. Importantly, the pretreatment procedure with ganetespib did not induce a cell cycle synchronization at the time of irradiation, excluding this uncertainty as a variable of radiosensitization. Upon irradiation, we observed a significant accumulation of sham-treated cells in S/G2/M phase within 8 h. This accumulation was consistently more pronounced in response to proton than in response to reference photon irradiation and resembles the similar but statistically insignificant trend as described in Hela and HSG cells by Iwata, et. al. [38]. In general, most cells shifted into G1 phase within 24 h after irradiation but a larger fraction of proton-irradiated cells persisted in S/G2/M phase. This difference was levelling out at the 48 h time point when the majority of irradiated cells accumulated in G1 phase. The presented elevated fraction of sham-treated cells in S/G2/M phase in response to proton irradiation suggests a more available respectively more utilized HRR. Interestingly, only in combination with irradiation, 10 nM ganetespib consistently decreased the fraction of cells in S/G2/M phase at each investigated time point. Reducing the fraction of cells in S/G2/M phase bears the potential to also reduce cellular access to HRR and as a consequence shift DNA repair pathway choice away from less erroneous HRR.

Our findings further demonstrated that protein levels of Rad51 are downregulated in irradiated A549 cells by 10 nM and also 2 nM ganetespib within 24 h. This indicates a HRR-suppressive role of ganetespib but may occur too slow to alter the initial cell cycle distribution or the early cellular response to radiation. Nonetheless, Rad51 downregulation in the days after irradiation still bears the potential to interfere with later cellular processes induced by the different radiation modalities, such as subsequent cell divisions, ultimately reducing clonogenicity.

Finally, we also investigated the emergence and removal of γH2AX foci in A549 cells as surrogate for DSBs in response to 2 Gy of ionizing irradiation. Similar numbers of foci were induced by photon and proton radiotherapy of both LETs as suggested by previous research [16]. In addition, also 24 h after irradiation the number of persisting foci was similar and independent of the nature of irradiation administered. Remarkably, despite the downregulation of Rad51 protein, neither γH2AX foci induction nor removal was altered in ganetespib-treated cells. These results deviate to findings obtained with more complete downregulation of Rad51 by siRNA which clearly delayed DSB foci removal at the 24 h time point [17]. Hence, Rad51 protein levels either remained sufficient in ganetespib-treated cells to accomplish HRR in the specified time or redundancies in DSB repair replaced the decreased capacity in Rad51-dependent HRR. As mentioned above, Rad51 downregulation by ganetespib was only observed at the 24 h time point and as a result potentially too late to alter the initial DNA DSB repair as investigated by this assay. In contrast, ganetespib could reduce clonogenicity by long time interference with the response during the cell division to misrepaired lesions and chromosomal aberrations initially induced by the different treatment modalities.

Due to the vast number of HSP90 client proteins, the exact mechanism of Rad51 downregulation upon low-dosed ganetespib treatment remains elusive. In detail, low-dosed ganetespib could directly affect the stability of Rad51 protein but could also affect the signalling upstream of Rad51 activation or expression. Moreover, alternative mechanisms of combinatorial sensitization by ganetespib affecting chaperone-mediated autophagy [39, 40], pro-tumorigenic signalling, or apoptosis [41, 42] were not investigated in this study. Importantly, the proton-specific sensitization as well as the reduction in Rad51 protein levels by HSP90 inhibition match previous findings generated with carbon irradiation [31–34] but are partially contradicted by findings in 3D pancreatic cancer cultures [43].

While NHEJ is indisputable the main DSB repair pathway in response to photon and proton irradiation [13, 43, 44], our findings, studies on SAHA [18], and recent studies employing DNA repair-deficient cell lines [45] support a differential cellular response to proton radiotherapy and an elevated importance of HRR. The broad range of cellular findings nevertheless requires a holistic approach to combine these variables and not exclude potential alterations in concomitant DNA repair proteins, cell cycle distribution, or unfolded protein response, to name a few.

## Conclusion

This study is the first to demonstrate radiosensitization for clinically relevant SOBP proton radiotherapy by HSP90 inhibition in cancer cells. We demonstrated that low-dosed HSP90 inhibitor ganetespib is sufficient to sensitize A549 cancer cell lines exclusively for proton radiotherapy but not for conventional photon irradiation. Corroborating findings could be observed in FaDu cells with similar increases in RBE of up to 30% present in both cell lines. Due to the narrow LET_D_ spectrum administered, no LET dependency of the radiosensitization by ganetespib could be established. As a result, pursuing research is required to establish the therapeutic window of LET-dependent drugging as devised by multiple studies for proton irradiation or shifting the combinatorial focus towards higher LET carbon irradiation.

## Supplementary Information

**Additional file 1**. **Table S1**Clonogenic cell survival - A549 & FaDu – α, β, and α/β. **Table S2** Clonogenic cell survival - A549 & FaDu – DMF & RBE. **Figure S1** Proton irradiation: instrumentation, phantom, and set-up

## Data Availability

Data is available upon request to the corresponding author.

## Abbreviations

ATCC: American Type Culture Collection
BrdU: Bromodeoxyuridine
CHO: Chinese hamster ovary
DAPI: 4’,6-Diamidin-2-phenylindol
DMF: Dose-modifying factor
DSB: Double strand break
HRP: Horseradish peroxidase
HRR: Homologous recombination repair
LET_D_: Dose-averaged linear energy transfer
NHEJ: Non-homologous end joining
PAGE: Polyacrylamide gel electrophoresis
PBS: Phosphate-buffered saline
PI: Propidium iodide
PMMA: Poly(methyl methacrylate), Plexiglas
PVDF: Polyvinylidene fluoride
RBE: Relative biological effectiveness
RPMI: Roswell Park Memorial Institute
SD: Standard deviation
SDS: Sodium dodecyl sulfate
SOBP: Spread-out Bragg peak
TBS-T: Tris-buffered saline with Tween 20
TPS: Treatment planning system
