# Supplementary material for: Ganetespib selectively sensitizes cancer cells for proximal and distal spread-out Bragg peak proton irradiation
Source: Radiat Oncol. 2022 Apr 11;17:72. doi: 10.1186/s13014-022-02036-z (PMC8996402; doi:10.1186/s13014-022-02036-z)
Supplement: Supplementary file 1 — Additional file 1. Table S1Clonogenic cell survival - A549 & FaDu – α, β, and α/β. Table S2 Clonogenic cell survival - A549 & FaDu – DMF & RBE. Figure S1 Proton irradiation: instrumentation, phantom, and set-up [file 13014_2022_2036_MOESM1_ESM.docx]

Deycmar et al., 2022 **Ganetespib selectively sensitizes cancer cells
for proximal and distal spread-out Bragg peak proton irradiation**

**Additional files**

**List of Content**

[Tab. S1 Clonogenic cell survival - A549 & FaDu – α, β, and α/β 1](#_Toc89083069)

[Tab. S2 Clonogenic cell survival - A549 & FaDu – DMF & RBE 2](#_Toc89083070)

[Fig. S1. Proton irradiation: instrumentation, phantom, and set-up 3](#_Toc89083071)

# Tab. S1 Clonogenic cell survival - A549 & FaDu – α, β, and α/β

Clonogenic cell survival data was fitted utilizing a linear quadratic model to obtain α and β. In sham-treated A549 cells, the fitted model shows a less linear response after proton irradiation than compared to photon irradiation. Ganetespib treatment of A549 cells linearized the response upon proton irradiation. FaDu cells follow a similar trend of a more linear response to proton irradiation upon ganetespib treatment.

| **A549** |  | **α** | **β** | **α/β** |  | **FaDu** |  | **α** | **β** | **α/β** |
| --- | --- | --- | --- | --- | --- | --- | --- | --- | --- | --- |
| **sham** | **photon** | **0.558** | **0.025** | **22.2** |  | **sham** | **photon** | **0.159** | **0.027** | **5.9** |
|  | **proximal SOBP** | **0.455** | **0.068** | **6.7** |  |  | **proximal SOBP** | **0.124** | **0.051** | **2.4** |
|  | **distal SOBP** | **0.422** | **0.090** | **4.7** |  |  | **distal SOBP** | **0.202** | **0.051** | **3.9** |
| **2nM Gan** | **photon** | **0.475** | **0.052** | **9.1** |  | **2nM Gan** | **photon** | **0.202** | **0.018** | **11.4** |
|  | **proximal SOBP** | **0.677** | **0.041** | **16.3** |  |  | **proximal SOBP** | **0.246** | **0.039** | **6.3** |
|  | **distal SOBP** | **0.653** | **0.060** | **11.0** |  |  | **distal SOBP** | **0.373** | **0.025** | **14.6** |

# Tab. S2 Clonogenic cell survival - A549 & FaDu – DMF & RBE

Clonogenic cell survival data of A549 and FaDu cells was fitted utilizing a linear quadratic model and DMF and RBE values were determined in triplicate at 50%, 25%, and 10% cell survival, respectively. To calculate DMF, sham-treated cells were compared to equally irradiated ganetespib-treated cells. For RBE, proton-irradiated cells were compared to equally treated cells irradiated with reference photons. p values were calculated by unpaired, two-tailed t-test with Welch’s Correction.

| **A549 - DMF  (gan vs. sham)** | | | **DMF_50_** | | | **DMF_25_** | | | **DMF_10_** | | |
| --- | --- | --- | --- | --- | --- | --- | --- | --- | --- | --- | --- |
|  |  |  | **mean** | **SD** | **p value^1^** | **mean** | **SD** | **p value^1^** | **mean** | **SD** | **p value^1^** |
| **photon** | | | **0.924** | **0.058** | **n.a.** | **0.970** | **0.028** | **n.a.** | **1.019** | **0.004** | **n.a.** |
| **proximal SOBP** | | | **1.322** | **0.048** | **0.0027 (**)** | **1.234** | **0.027** | **0.0013 (**)** | **1.164** | **0.014** | **0.0031 (**)** |
| **distal SOBP** | | | **1.327** | **0.123** | **0.0359 (*)** | **1.226** | **0.061** | **0.0221 (*)** | **1.151** | **0.020** | **0.0078 (**)** |
| **^1^compared to reference photon irradiation** | | | | | | | | | | | |
|  |  | |  |  |  |  |  |  |  |  |  |
| **A549 - RBE**  **(proton vs. photon)** | | | **RBE_50_** | | | **RBE_25_** | | | **RBE_10_** | | |
| **treatment** | | | **mean** | **SD** | **p value** | **mean** | **SD** | **p value** | **mean** | **SD** | **p value** |
| **proximal SOBP** | | **sham** | **0.928** | **0.052** | **0.0272 (*)** | **0.994** | **0.031** | **0.0267 (*)** | **1.068** | **0.049** | **0.0368 (*)** |
| **proximal SOBP** | | **2nM** | **1.33** | **0.105** |  | **1.265** | **0.072** |  | **1.214** | **0.051** |  |
| **distal SOBP** | | **sham** | **0.921** | **0.108** | **0.0196 (*)** | **1.014** | **0.049** | **0.0127 (*)** | **1.112** | **0.039** | **0.0299 (*)** |
| **distal SOBP** | | **2nM** | **1.318** | **0.105** |  | **1.281** | **0.071** |  | **1.250** | **0.047** |  |
|  |  | |  |  |  |  |  |  |  |  |  |
| **FaDu - DMF  (gan vs. sham)** | | | **DMF_50_** | | | **DMF_25_** | | | **DMF_10_** | | |
|  |  |  | **mean** | **SD** | **p value^1^** | **mean** | **SD** | **p value^1^** | **mean** | **SD** | **p value^1^** |
| **photon** | | | **1.057** | **0.018** | **n.a.** | **0.998** | **0.012** | **n.a.** | **0.960** | **0.008** | **n.a.** |
| **proximal SOBP** | | | **1.263** | **0.026** | **0.0015 (**)** | **1.153** | **0.013** | **0.0006 (***)** | **1.088** | **0.012** | **0.0006 (***)** |
| **distal SOBP** | | | **1.342** | **0.054** | **0.0130 (*)** | **1.191** | **0.003** | **0.0013 (**)** | **1.090** | **0.034** | **0.0237 (*)** |
| **^1^compared to reference photon irradiation** | | | | | | | | | | | |
|  |  | |  |  |  |  |  |  |  |  |  |
| **FaDu - RBE**  **(proton vs. photon)** | | | **RBE_50_** | | | **RBE_25_** | | | **RBE_10_** | | |
| **treatment** | | | **mean** | **SD** | **p value** | **mean** | **SD** | **p value** | **mean** | **SD** | **p value** |
| **proximal SOBP** | | **sham** | **1.094** | **0.040** | **0.0078 (**)** | **1.162** | **0.011** | **0.0002 (***)** | **1.205** | **0.036** | **0.0104 (*)** |
| **proximal SOBP** | | **1nM** | **1.308** | **0.042** |  | **1.343** | **0.007** |  | **1.367** | **0.032** |  |
| **distal SOBP** | | **sham** | **1.324** | **0.039** | **0.0217 (*)** | **1.334** | **0.031** | **0.0020 (**)** | **1.343** | **0.069** | **0.0531 (n.s.)** |
| **distal SOBP** | | **1nM** | **1.682** | **0.087** |  | **1.592** | **0.030** |  | **1.523** | **0.030** |  |

# **Fig. S1.** Proton irradiation: instrumentation, phantom, and set-up


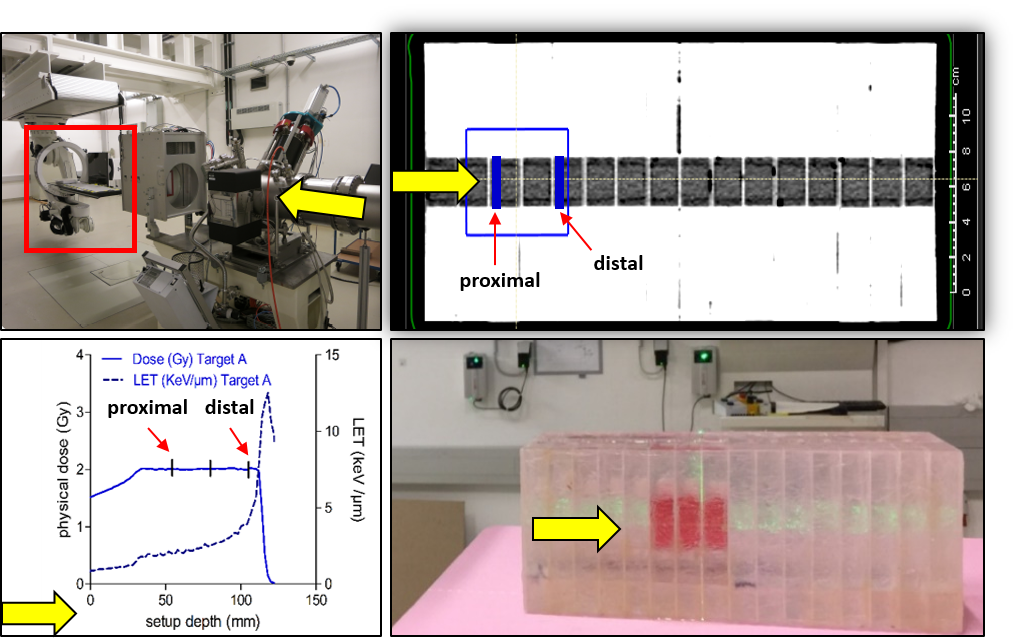


**B**

**A**

**C**

**D**

(A) Fixed horizontal proton beam line. The phantom is placed on the robotic patent stage as marked with a red box. Beam direction is marked by a bright yellow arrow. (B) CT image (top view) of the PMMA/water phantom. The blue box illustrates the planned treatment field and the blue lines the position of the cell layers in the treatment field as indicated by red arrows. (C) Dose/LET simulation of the administered SOBP. The respective depths of the cell layers in the SOBP are indicated by red arrows and the corresponding dose-averaged LET illustrated by the dashed line. (D) Accurate and reproducible alignment of the PMMA/water phantom was assured by positioning lasers in the treatment room.
